# Menstrual blood-derived stromal cells modulate functional properties of mouse and human macrophages

**DOI:** 10.1038/s41598-020-78423-x

**Published:** 2020-12-07

**Authors:** Rocío Martínez-Aguilar, Salvador Romero-Pinedo, M. José Ruiz-Magaña, Enrique G. Olivares, Carmen Ruiz-Ruiz, Ana C. Abadía-Molina

**Affiliations:** 1 Unidad de Inmunología, IBIMER, CIBM, Universidad de Granada, Granada, Spain; 2 Departamento de Bioquímica y Biología Molecular III e Inmunología, Facultad de Medicina, Universidad de Granada, Granada, Spain; 3 Unidad de Gestión Clínica Laboratorios, Hospital Universitario Clínico San Cecilio, Granada, Spain

**Keywords:** Immunology, Stem cells

## Abstract

Menstrual blood-derived stromal cells (MenSCs) are emerging as a strong candidate for cell-based therapies due to their immunomodulatory properties. However, their direct impact on innate immune populations remains elusive. Since macrophages play a key role in the onset and development of inflammation, understanding MenSCs implication in the functional properties of these cells is required to refine their clinical effects during the treatment of inflammatory disorders. In this study, we assessed the effects that MenSCs had on the recruitment of macrophages and other innate immune cells in two mouse models of acute inflammation, a thioglycollate (TGC)-elicited peritonitis model and a monobacterial sepsis model. We found that, in the TGC model, MenSCs injection reduced the percentage of macrophages recruited to the peritoneum and promoted the generation of peritoneal immune cell aggregates. In the sepsis model, MenSCs exacerbated infection by diminishing the recruitment of macrophages and neutrophils to the site of infection and inducing defective bacterial clearance. Additional in vitro studies confirmed that co-culture with MenSCs impaired macrophage bactericidal properties, affecting bacterial killing and the production of reactive oxygen intermediates. Our findings suggest that MenSCs modulate the macrophage population and that this modulation must be taken into consideration when it comes to future clinical applications.

## Introduction

Menstrual blood-derived stromal cells (MenSCs) are considered a type of mesenchymal stromal cells (MSCs), originally located in the endometrium, that can be isolated from menstrual fluid. MenSCs share characteristics with other MSCs such as being plastic-adherent cells, expressing typical mesenchymal stromal cell surface markers, being capable of differentiating into mesodermal cell lineages in vitro and displaying a low immunogenic profile^1,2^, which makes them a good candidate for clinical use in cell-based therapies.

Since their first characterisation by Meng et al.^1^ in 2007, MenSCs have aroused great interest as an alternative source of MSCs other than bone marrow^3^, adipose tissue^4^ or birth-associated tissues—such as umbilical cord^5^, cord blood^6^, decidua^7^ and placenta^8^—due to their ease of obtainment. In contrast to other tissue sources, MenSCs collection from menstrual fluid is non-invasive, allows the isolation of a large number of cells and it is exempted from ethical dilemmas. All those properties have boosted their use in several pre-clinical models^9^ and some early-stage clinical trials^10^.

The initial use of bone marrow and adipose-derived MSCs has generated extensive literature regarding the therapeutic role of MSCs. Their actions are known to be mediated by paracrine mechanisms, which provide a myriad of pleiotropic effects including angiogenic, anti-apoptotic and immunomodulatory properties^11,12^. Among all the effects displayed, MSCs ability to modulate the immune system stands as a promising tool for the treatment of inflammation^13,14^.

The immunomodulatory effects of MSCs are mainly dependent on the release of cytokines and anti-inflammatory factors, although cell–cell contact through the action of different ligands has also been described^15–17^. Since the tissue source is a factor that might contribute to the particular functional properties and effects exerted by MSCs, one must be cautious when extrapolating the immunomodulatory properties of MSCs from different origins^2,18–21^. Bearing this in mind, it becomes necessary to understand the specific mechanisms of action by which MenSCs modulate immune populations.

Several studies have described MenSCs effects in different models of inflammation such as experimental colitis^22,23^, lipopolysaccharide (LPS)-induced injury^24,25^ or polymicrobial sepsis^26^. However, these studies are focused on the systemic outcome of the treatment—e.g. survival rates or reduction of histopathological damage—or the direct impact of MenSCs on T and B cell populations.

Macrophages are essential mediators of the innate immune response and play a crucial role in the development and maintenance of inflammation^27^. To date, the impact of MenSCs on macrophage function is unknown. To better aid clinical translation of MenSCs, unravelling their overall effect in the inflammatory process is needed. Therefore, studies of the impact of MenSCs on key players of inflammation, such as macrophages, could substantially contribute to understand their mode of action during the treatment of inflammatory disorders.

The purpose of this study was to evaluate the immunomodulatory effects that MenSCs have on macrophages during the early stages of acute inflammation using two mouse models: a thioglycollate (TGC)-elicited peritonitis model and a monobacterial sepsis model induced by *Salmonella* Typhimurium strains. We analysed the effect that the presence of MenSCs had on the recruitment of macrophages and other innate immune cells—such as neutrophils and inflammatory monocytes—and measured general parameters to monitor the inflammatory status of the mice. In addition, we studied the direct effect that MenSCs had on human macrophage functional properties, such as phagocytosis and bacterial clearance through the production of oxygen reactive intermediates.

## Results

### Injection of MenSCs alters the number and percentage of innate immune populations recruited to the peritoneum in a TGC-elicited peritonitis mouse model

**Figure 1:**
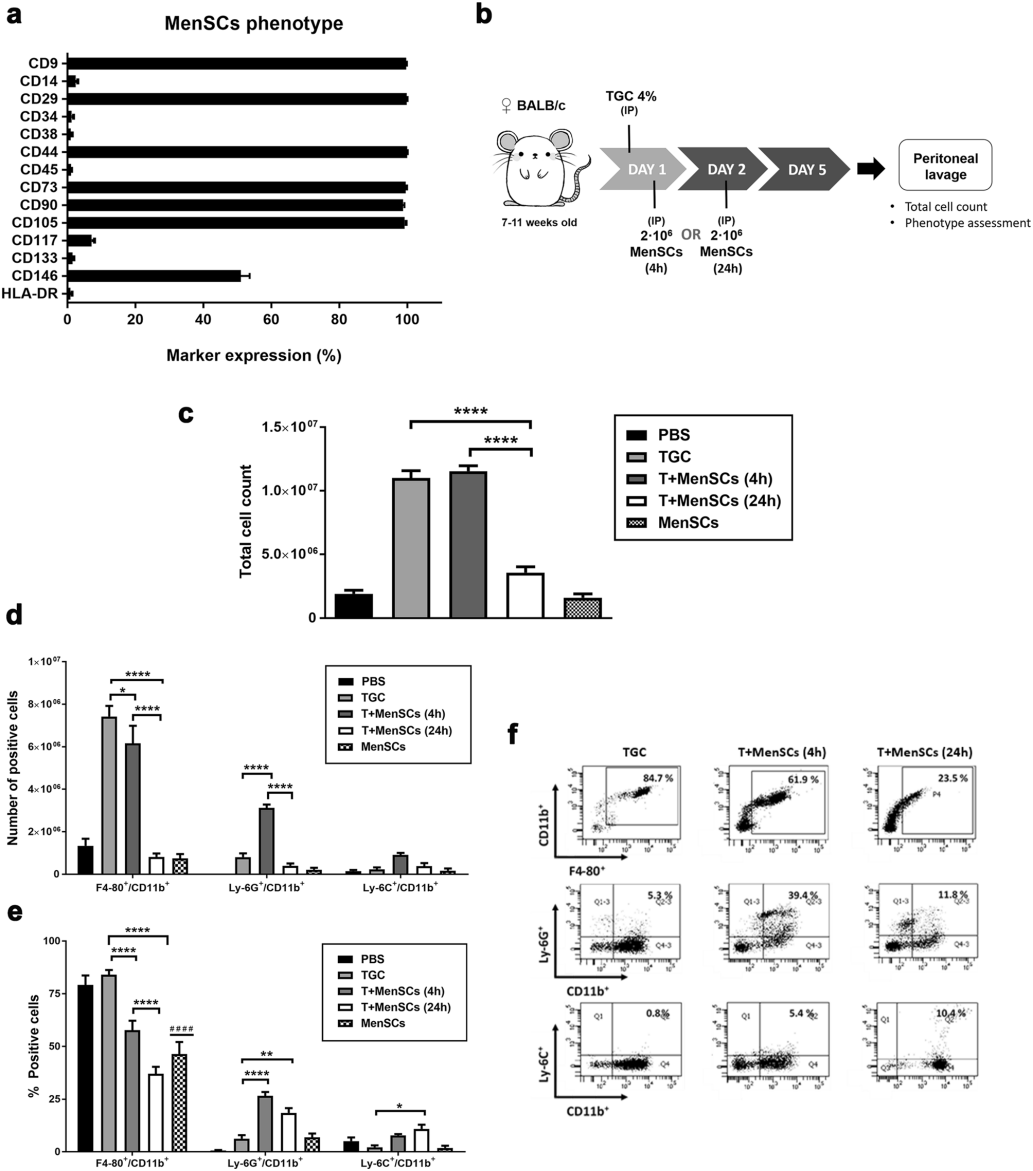
Effects of MenSCs in a TGC-elicited peritonitis mouse model. (**a**) MenSCs phenotype assessment by flow cytometry. (**b**) Overview of mouse model TGC-elicited peritonitis and MenSCs treatment. (**c**) Total number of cells recruited in the peritoneal cavity 4 days after TGC administration. (**d**,**e**) Phenotypic characterisation of the cells recovered from the peritoneal lavage. Macrophage population was gated as F4-80^+^/CD11b^+^, neutrophils as Ly-6G^+^/CD11b^+^ and inflammatory monocytes as Ly-6C^+^/CD11b^+^. Results are represented as mean ± SEM (n = 4–9 mice per experimental group)**.** (**f**) Representative flow cytometry analysis of mice peritoneal lavage from TGC, T + MenSCs (4 h) and T + MenSCs (24 h) experimental groups. All experiments were performed using 4 different MenSCs donors. *p < 0.05, **p < 0.01, ***p < 0.001, ****p < 0.0001. Statistical significance compared to PBS group (#). *IP* intraperitoneal injection, *T + MenSCs* TGC + MenSCs administration mice group.

Firstly, we confirmed that the isolated MenSCs were equivalent to those previously reported^1,28^ by comparing morphological and phenotypic characteristics. Freshly isolated MenSCs exhibited a fibroblast-like shape when cultured on plastic (Fig. S1), were able to differentiate in vitro into osteoblasts, adipocytes and chondroblasts (data not shown), and showed a characteristic MSCs surface marker phenotype (Fig. 1a). They were positive for commonly identified MSC surface markers such as CD9, CD29, CD44, CD73, CD90, CD105 and CD146, exhibited a low expression of the bone marrow progenitor cell marker CD117 and were negative for hematopoietic and endothelial markers including CD14, CD34, CD38, CD45, CD133 and HLA-DR. All the characteristics previously described make MenSCs meet minimal criteria to be considered human MSCs^29^.

MSCs have been described as immune response modulators, showing differential effects depending on their tissue source^2,18–21^. To evaluate the direct impact that MenSCs have on innate immune cells recruitment at the onset of inflammation, we administered MenSCs into a TGC-elicited peritonitis mouse model. In this model, TGC acts as an eliciting agent responsible for recruiting a large number of innate immune cells to the site of injection^30^. The influx of neutrophils reach peak levels between 4 and 24 h after TGC injection, while macrophage start their recruitment at 24 h and reach peak levels at 3–4 days^31–33^. MenSCs were intraperitoneally (IP) administered at either 4 h or 24 h after TGC injection; as controls, PBS, TGC alone or MenSCs alone were used (Fig. 1b). At day 5, mice were sacrificed, and peritoneal lavages were recovered to characterise the number and phenotype of recruited cells (Fig. 1c–f).

The number of cells recruited when MenSCs were administered 4 h after TGC treatment (T-MenSC-4 h) was similar to that of the TGC control group (1.1 × 10^7^ ± 4.2 × 10^5^ and 1.1 × 10^7^ ± 5.6 × 10^5^ respectively), while MenSCs injected at 24 h (T-MenSC-24 h) significantly reduced the total number of cells recruited (3.5 × 10^6^ ± 4.7 × 10^5^, p < 0.0001) (Fig. 1c). Moreover, recruited cell numbers were not affected by administration of MenSCs alone compared to the PBS group (1.8 × 10^6^ ± 3 × 10^5^ and 1.8 × 10^6^ ± 2.8 × 10^5^ respectively).

To fully characterise peritoneal recruited cells, their phenotype was assessed by flow cytometry. Macrophages (F4-80^+^/CD11b^+^), the main population of immune cells recruited in the TGC experimental conditions (7.4 × 10^6^ ± 5 × 10^5^ cells), were significantly reduced in T-MenSC-4 h (6.1 × 10^6^ ± 8.2 × 10^5^ cells, p < 0.01) and dramatically reduced in T-MenSC-24 h (8.2 × 10^5^ ± 1.5 × 10^5^ cells, p < 0.0001) mice compared to single TGC group (Fig. 1d). In addition, while T-MenSC-24 h and single TGC group had a similar number of neutrophils (Ly-6G^+^/CD11b^+^) (4 × 10^5^ ± 1 × 10^5^ cells and 8 × 10^5^ ± 1.7 × 10^5^ respectively), T-MenSC-4 h showed a statistically significant increment of cells for this population (3.1 × 10^6^ ± 1.5 × 10^5^ cells, p < 0.0001) (Fig. 1d). Regarding the absolute number of inflammatory monocytes (Ly-6C^+^/CD11b^+^), no significant differences were observed between any of the experimental groups (Fig. 1d).

To determine whether the proportion of each immune population recruited was affected by MenSCs administration, the percentage of cells was also studied (Fig. 1e,f). We observed a progressive reduction of the percentage of macrophages in T-MenSC-4 h (58% ± 4.4, p < 0.0001) and T-MenSC-24 h (37% ± 3.3, p < 0.0001) compared to the TGC group (84% ± 2.3), while the neutrophil percentages increased significantly in both groups, T-MenSC-4 h (26% ± 1.8, p < 0.0001) and T-MenSC-24 h (18% ± 2.2, p < 0.01), in comparison to the TGC group (6% ± 1.6) (Fig. 1e,f). The percentage of inflammatory monocytes (Ly-6C^+^/CD11b^+^) in T-MenSC-24 h group was significantly increased (11% ± 1.9, p < 0.05) compared to the TGC group (2% ± 0.8) (Fig. 1e,f). Notably, when MenSCs were administered alone the percentage of macrophages recruited (46% ± 5.7, p < 0.0001) was significantly reduced compared to the PBS group (80% ± 4.5) (Fig. 1e).

Interestingly, while collecting the peritoneal lavage cells, a nodular structure was observed in the mice peritoneum of the T-MenSC-24 h injection group, which was subsequently analysed.

### Characterisation of the peritoneal aggregates generated after TGC + MenSCs injection in mice

**Figure 2:**
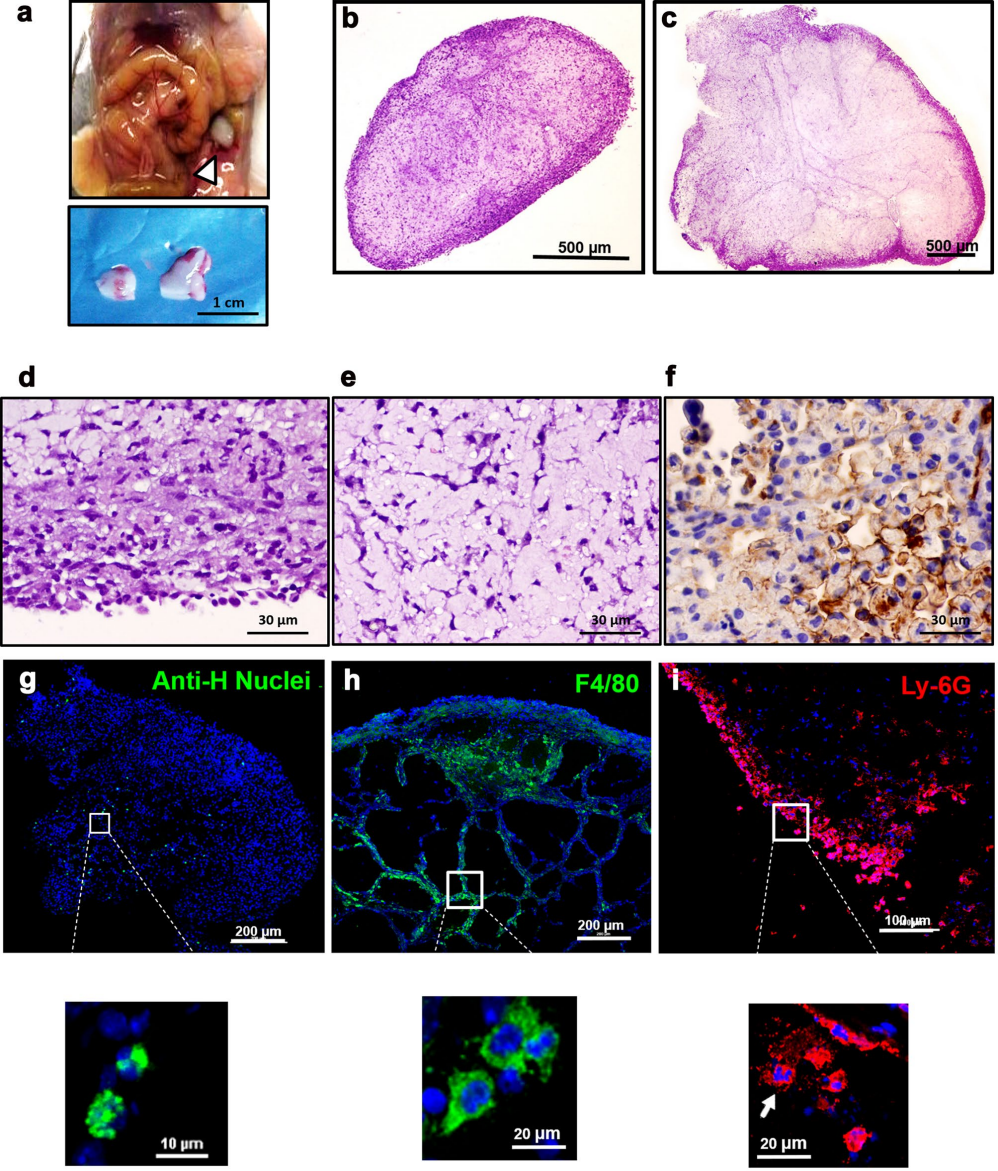
Histopathological evaluation of the nodule generated after T + MenSCs administration. (**a**) Representative image of the nodule. (top, arrowhead) Peritoneum location 3 days after MenSCs administration under the TGC inflammatory insult. (bottom) Nodules recovered from mice injected with TGC + MenSCs-24 h. (**b**–**f**) Representative images of nodules taken by optical microscopy. (**b**) Longitudinal and (**c**) cross H&E stained sections from a paraffin embedded sample*.* 20 × magnification of the (**d**) peripheral and (**e**) central area of the structure*.* (**f**) Human cytokeratin^+^ cells within the aggregates. (**g**) MenSCs, (**h**) macrophage and (**i**) neutrophil populations distributed within the OCT-frozen structure as determined by laser confocal microscopy after staining with the appropriate antibodies: (**g** anti-human nuclei^+^-AF488; **h** anti-F4/80^+^-AF488; **i** anti-Ly-6G^+^-AF594). A total of 3 different nodules were histopathologically characterised.

Aggregates observed during the peritoneal exam of T-MenSCs-24 h mice were subjected to macroscopic evaluation, cryosectioned and analysed by optical and confocal microscopy (Fig. 2). Macroscopically, they could be defined as solid, white nodules of considerable size (0.5–1 cm) (Fig. 2a). The H&E stained sections showed no sign of necrosis and revealed a mixed cell population (Fig. 2b–e). In particular, an accumulation of cells was observed at the periphery of the structure, whereas the central zone was occupied by a lax stroma (fat). At higher magnifications, polymorphonuclear cells were identified peripherally (Fig. 2d,e), while a population of human cytokeratin^+^ cells was found within the stromal compartment (Fig. 2f).

Confocal analysis displayed a uniform distribution of anti-human nuclei^+^ cells throughout the nodule (Fig. 2g). Macrophages (F4/80^+^) were identified as the main cellular component, forming clusters within the internal core as well as on the outer surface of the nodular structure (Fig. 2h). Neutrophils (Ly-6G^+^) were also present, limiting their location to the periphery (Fig. 2i) as previously suggested by the H&E sections (Fig. 2d).

### MenSCs exacerbate *Salmonella* Typhimurium infection in a sepsis mouse model

**Figure 3:**
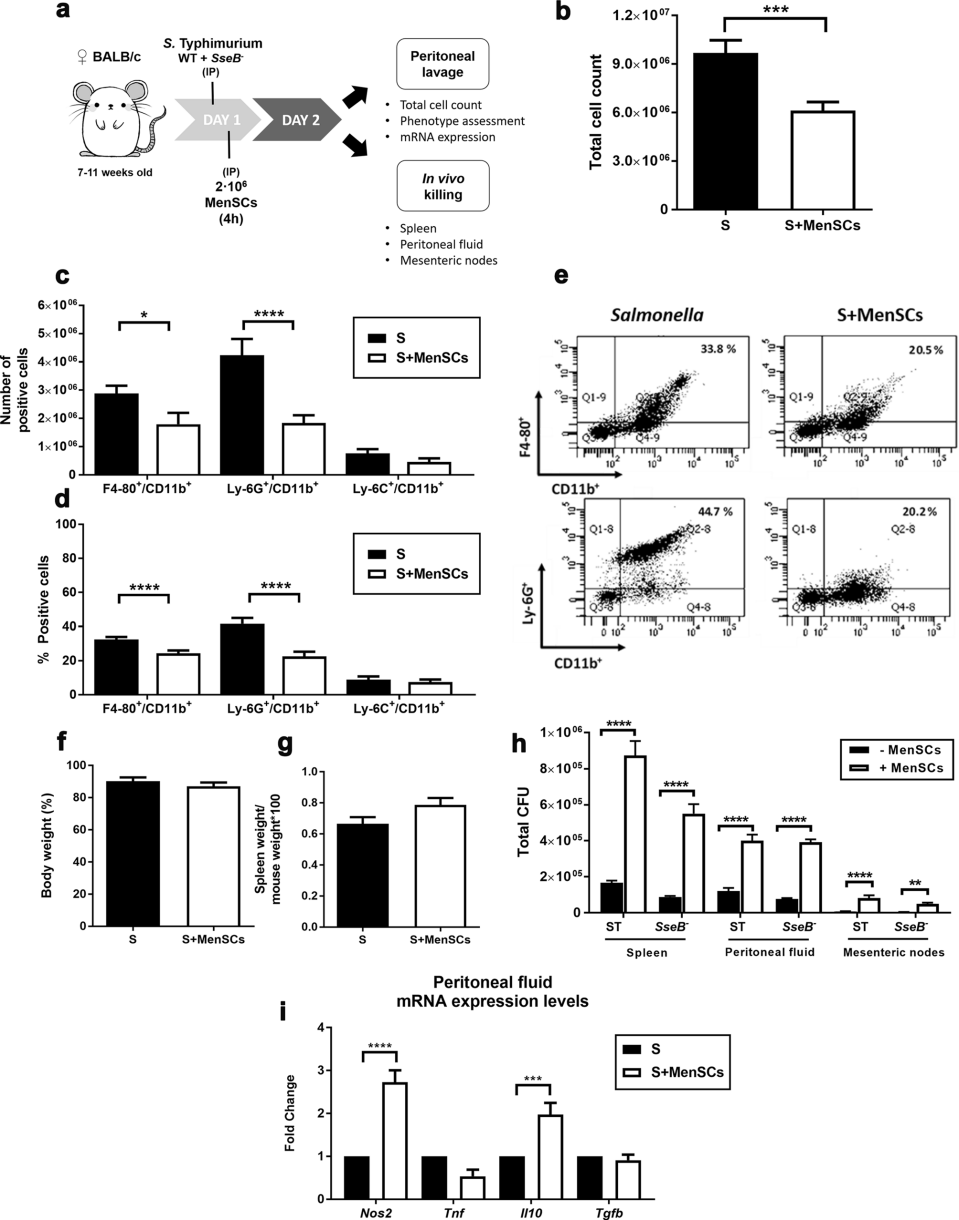
Effects of MenSCs in a sepsis mouse model. (**a**) Overview of a sepsis mouse model induced by *S.* Typhimurium injection and MenSCs treatment. (**b**) Total number of cells recruited in the peritoneal cavity 24 h after MenSCs administration. (**c,d**) Phenotypic characterisation of the cells recovered from the peritoneal lavage. Macrophage population was gated as F4-80^+^/CD11b^+^, neutrophils as Ly-6G^+^/CD11b^+^ and inflammatory monocytes as Ly-6C^+^/CD11b^+^. (**e**) Representative flow cytometry analysis of mice peritoneal lavage from *Salmonella* and S + MenSCs experimental groups. (**f**) Mice body weight represented as [weight before sacrifice/initial weight] × 100. (**g**) Spleen weight relativised to the mice body weight before sacrifice. (**h**) Bacterial load in spleen, peritoneal fluid and mesenteric nodes obtained from *Salmonella*-infected mice treated with and without MenSCs. (**i**) Relative mRNA expression of *Nos2*, *Tnf*, *Il10* and *Tgfb* in peritoneal fluid lysates referred to mRNA expression levels in infected mice without MenSCs injection. Results are represented as mean ± SEM (n = 12–13 mice per experimental group). All experiments were performed using 4 different MenSCs donors. *p < 0.05, **p < 0.01, ***p < 0.001, ****p < 0.0001. *IP* intraperitoneal injection, *S* Salmonella (ST + SseB^*−*^)-infected mice**,** *CFU* colony-forming units.

We further explored the effects of MenSCs in a model of systemic typhoid-like disease^34^ caused by *Salmonella enterica* Serovar Typhimurium (*Salmonella*) infection in susceptible mice. In this model, a mix of wild type (ST) and attenuated (*SseB*^*−*^)^35^ *Salmonella* strains are co-injected (Fig. 3a). MenSCs were IP administered 4 h after the injection of *Salmonella*, that is, at the onset of sepsis^36^ (Fig. 3a).

Twenty-four hours after sepsis induction, the peritoneal cell count of *Salmonella* + MenSCs mice showed a significant reduction in the influx of cell populations (6.1 × 10^6^ cells ± 5.2 × 10^5^, p < 0.001) compared to *Salmonella* group (9.7 × 10^6^ ± 7.9 × 10^5^) (Fig. 3b). In this control *Salmonella* group, the main immune populations comprised of neutrophils (Ly-6G^+^/CD11b^+^) (4.2 × 10^6^ ± 5.7 × 10^5^) followed by macrophages (F4-80^+^/CD11b^+^) (2.9 × 10^6^ ± 2.7 × 10^5^) (Fig. 3c). In contrast, the *Salmonella* + MenSCs group showed similar numbers of macrophages and neutrophils (1.8 × 10^6^ ± 4 × 10^5^ and 1.8 × 10^6^ ± 2.7 × 10^5^ respectively), being both populations significantly fewer in number compared to *Salmonella* group (p < 0.05, p < 0.001 respectively) (Fig. 3c). As for inflammatory monocytes (Ly-6C^+^/CD11b^+^), no significant changes in cell number were detected between groups (Fig. 3c).

Analyses of the percentage populations also showed a statistically significant reduction in the percentage of both neutrophils (22.5% ± 2.7, p < 0.0001) and macrophages (24% ± 1.6, p < 0.0001) compared to the control *Salmonella* group (42% ± 3.3 and 32% ± 1.5 respectively), being consistent with the reduction in number of positive cells (Fig. 3d,e). Regarding the percentage of inflammatory monocytes, no significant differences were detected between experimental groups (Fig. 3d). Interestingly, while using the *Salmonella* model, no peritoneal cell aggregation was detected during the collection of the peritoneal lavage cells.

To evaluate the septic state of the mice, we monitored the weight loss (Fig. 3f), spleen inflammation (Fig. 3g) and colony-forming unit (CFU) burden in infected organs and tissues such as the spleen, mesenteric nodes and peritoneal fluid (Fig. 3h). Although no differences in body and spleen weight were found between *Salmonella* and *Salmonella* + MenSCs experimental groups, the bacterial load in MenSCs-injected mice increased remarkably in the spleen compared to the *Salmonella* group indicating significantly inferior bacteria clearance (Fig. 3h, see Table S1) and suggesting an increment in the proliferative activity of the bacteria^35,37^. This pattern was also observed for peritoneal fluid and mesenteric nodes (Fig. 3h, see Table S1).

During early sepsis, there is an intense production of pro-inflammatory cytokines which leads to multiple organ failure and eventually death^36^. To give insight into the peritoneal inflammatory context, levels of mRNA expression of pro- (inducible nitric oxide synthase (*Nos2*), tumour necrosis factor-α (*Tnf*)) and anti-inflammatory mediators (*Il10*, *Tgfb*) were also analysed (Fig. 3i). *Nos2* expression was threefold upregulated in the peritoneal fluid of *Salmonella* + MenSCs mice compared to *Salmonella* mice. This pattern was also found in *Il10* expression (twofold increase) whereas no changes were observed in *Tgfb* and *Tnf* mRNA levels.

### Co-culture with MenSCs results in bacterial killing impairment of the human macrophage cell line THP-1

The previous results derived from the sepsis mouse model suggest that the role of macrophages in this bacterial infection could be modulated by MenSCs. To decipher whether MenSCs may affect bacterial clearance capability of human macrophages we evaluated the phenotype, phagocytic and killing capacity of PMA-differentiated THP-1 (THP-1_PMA_) cells in vitro.

**Figure 4:**
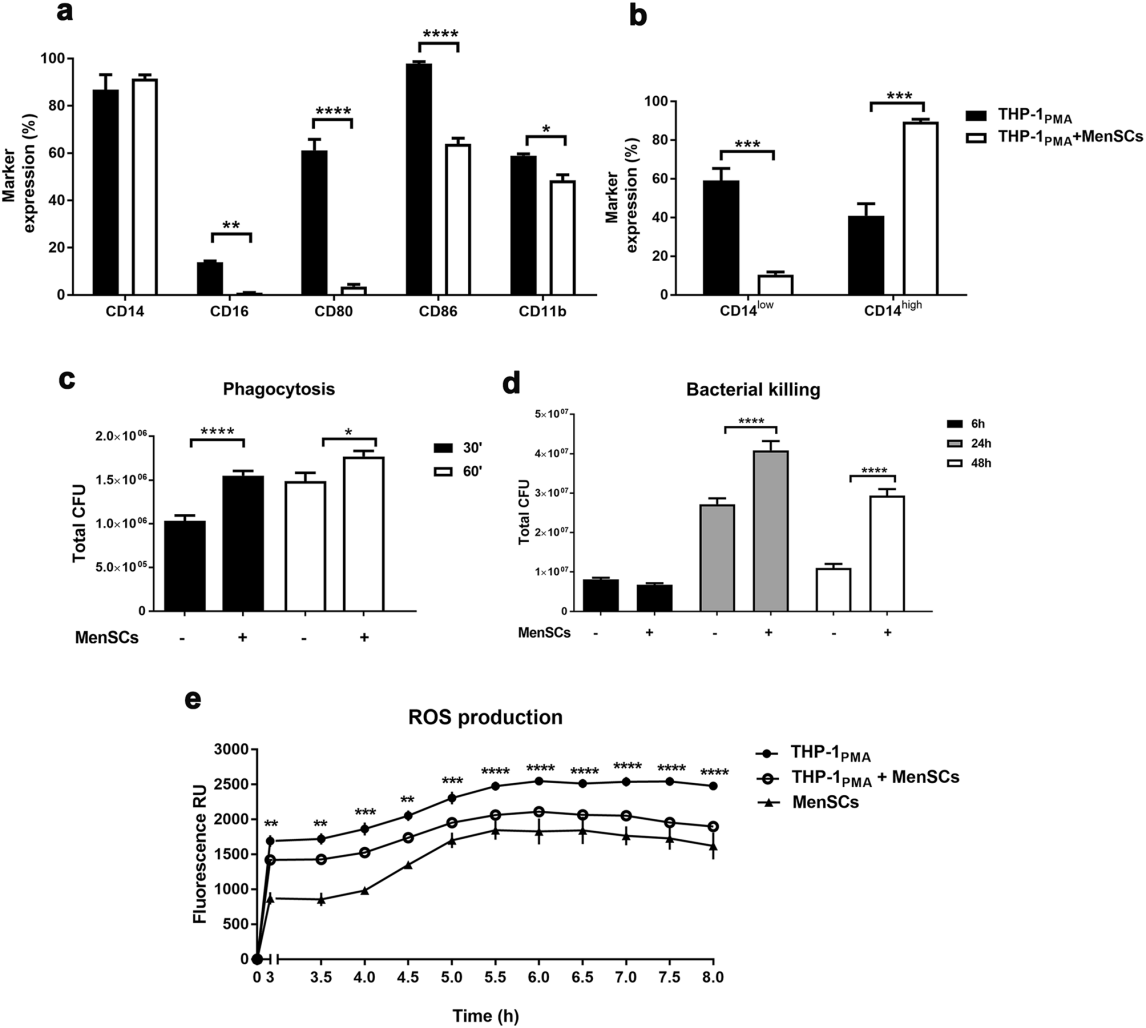
Evaluation of the activity of PMA-differentiated THP-1 cells co-cultured with MenSCs. PMA-differentiated THP-1 cells (THP-1_PMA_) were co-cultured or not with MenSCs (ratio 4:1) for 48 h (**c**–**e**) or 72 h (**a,b**). (**a**) Phenotypic characterisation and (**b**) CD14 specific expression pattern of THP-1_PMA_. (**c**) THP-1_PMA_ intake of *Salmonella SseB*^*−*^ after 30 min and 60 min of bacteria exposure. (**d**) Gentamicin protection assay reflecting bacteria killing time lapse of THP-1_PMA_ after exposure to *Salmonella SseB*^*−*^ for 60 min. (**e**) ROS production in vitro was measured by DCFDA oxidation. Results are represented as mean ± SEM of 3–6 different MenSCs, derived from different donors (n = 3–10 replicates). *p < 0.05, **p < 0.01, ***p < 0.001, ****p < 0.0001. *RU* relative units, *CFU* colony-forming units.

First, we co-cultured THP-1_PMA_ with MenSCs for 72 h and analysed the surface expression of macrophage markers (CD14, CD16, CD80, CD86 and CD11b) to determine whether their differentiation state could be affected by MenSCs (Fig. 4a). Co-cultured THP-1_PMA_ showed a significant decrease in the expression of the co-stimulatory molecules CD80 (17-fold reduction, p < 0.0001) and CD86 (1.5-fold reduction, p < 0.0001), the marker for classical macrophages CD16 (15-fold reduction, p < 0.01), and the monocyte-to-macrophage differentiation marker CD11b (1.2-fold reduction, p < 0.05), suggesting a switch to a more regulatory phenotype (Fig. 4a). Moreover, co-culture with MenSCs increased THP-1_PMA_ CD14 surface expression (twofold increase in the number of CD14^high^ cells, p < 0.001) compared to THP-1_PMA_ cultured alone (Fig. 4b).

To evaluate the phagocytic capacity of THP-1_PMA_ co-cultured with MenSCs (ratio 4:1) for 48 h, they were then challenged with *SseB*^*−*^ for 30 and 60 min. MenSCs co-culture significantly increased the phagocytic capacity of THP-1_PMA_ compared to THP-1_PMA_ cultured alone (*30* min 1.5-fold increase p < 0.0001, *60* min 1.1-fold increase p < 0.05) (Fig. 4c).

In order to measure macrophage effectiveness in blocking *Salmonella* intracellular replication, we performed a gentamicin protection assay at 6, 24 and 48 h (Fig. 4d). Six hours after the bacteria challenge, the number of viable bacteria recovered from THP-1_PMA_ co-cultured with MenSCs was similar to that without co-culture treatment. However, at both 24 h and 48 h after *SseB*^*−*^ exposure, there was a significant increase of bacteria recovered from THP-1_PMA_ co-cultured with MenSCs compared to THP-1_PMA_ cultured alone (24 h 1.5-fold increase p < 0.0001, 48 h 2.6-fold increase p < 0.0001) (Fig. 4d), indicating that MenSCs severely impaired the bacteria killing ability of THP-1_PMA_, and favoured *Salmonella* proliferation^37^.

To further evaluate whether the inefficient microbicide effects were due to a defective respiratory burst, reactive oxygen species (ROS) production was measured (Fig. 4e). DCFDA oxidation measurement showed a significant reduction in ROS production by THP-1_PMA_ directly co-cultured with MenSCs at all time points compared to THP-1_PMA_ cultured alone. Hence, in the context of in vitro* SseB*^*−*^ infection, MenSCs might diminish ROS production in human THP-1_PMA_ and as a consequence bacteria clearance is reduced. Interestingly, THP-1_PMA_ ROS production was diminished even though MenSCs primed with *SseB*^*−*^ were also able to generate microbicidal oxygen intermediates (Fig. 4e).

## Discussion

The clinical potential of MenSCs is undeniable. From a technical perspective, their non-invasive collection and exemption from ethical concerns are advantages that make MenSCs good alternatives to other MSCs sources. On the other hand, from a therapeutic perspective, MenSCs general immunomodulatory properties^1,2^ are a promising feature to explore for future cell-based therapies, especially for those involving the treatment of immune disorders. However, the extent of their modulatory effects on inflammation remain poorly understood. To further unravel their properties, we analysed MenSCs and macrophage crosstalk in two different models of acute peritonitis, a *germ free* TGC-elicited peritonitis model and a monobacterial sepsis model induced by *Salmonella* Typhimurium strains. Each model displays a particular inflammatory context, which allows us to study MenSCs impact on macrophages in a tightly controlled setting.

We found that, when MenSCs were injected 4 or 24 h after the TGC administration, the number and percentage of macrophages recruited to the peritoneum decreased. Interestingly, this reduction was more evident on the T-MenSCs-24 h mice group where it correlated with the appearance of peritoneal aggregates. It is not the first time that peritoneal aggregates are found after the administration of MSCs. Using a colitis mouse model, Sala et al.^38^ found that mouse bone marrow and human adipose MSCs formed aggregates in the peritoneal cavity when IP injected in mice^38^. Considering the ability of MenSCs to identify sites of injury and direct their migration towards them^26,39,40^, we hypothesise that MenSCs injected at the 24 h time point identify the local inflammatory milieu and use the peritoneal adipose tissue—close to the site of injection—as a scaffold to recruit and modulate peritoneal cell populations. The internal organisation of the aggregates seems to support this hypothesis, where MenSCs are located within the structure whereas macrophages can be found peripherally, as if they were later recruited.

The distribution of macrophages within the peritoneal aggregates also suggest bloodstream recruitment, as they could be found clustered surrounding the adipose tissue blood vessels. Although further studies are needed, both the systemic and local attraction could be mediated by the chemokine CCL2, which is responsible for recruiting monocytes and macrophages^14^ and has been found to be constitutively expressed by MenSCs^41^.

The presence of MenSCs in the TGC-elicited peritonitis also affected the influx of other innate immune populations. For instance, the percentage of neutrophils recruited towards the peritoneum, both at 4 h and 24 h hours after TGC administration, was significantly increased. Likewise, neutrophils were also found in the peritoneal aggregates, suggesting that MenSCs might promote local neutrophil recruitment. Although the specific impact of MenSCs on neutrophils is outside the scope of this study, it would be very informative to address whether MenSCs can modulate neutrophil lifespan and chemotaxis.

In the monobacterial sepsis model, the administration of MenSCs reduced the number of phagocytes—macrophages and neutrophils—recruited to the peritoneum. To determine whether the diminished microbicidal activity observed in the peritoneal fluid was not only due to the macrophage cell number but also to their phenotype, we analysed a set of classical pro- (*Nos2*, *Tnf*) and anti-inflammatory mediators (*Il10*, *Tgfb*). The upregulation of *Nos2* and *Il10* mRNA levels in the peritoneal fluid of MenSCs-treated mice suggests that MenSCs may promote a regulatory macrophage phenotype. Regulatory macrophages are known to possess an hybrid phenotype which shares M1 (NOS2) and M2 (IL-10, arginase-1) macrophage classical markers, with the main function of reestablishing tissue homeostasis^42–45^. They also display poor bactericidal properties^42^ mainly due to IL-10 expression^46^. Furthermore, the secretion of IL-10 by regulatory macrophages can prevent neutrophils migrating into the inflamed tissue^47^, supporting the significant decrease in neutrophil recruitment after MenSCs injection. Far from considering a contradiction the apparently opposite results regarding neutrophil recruitment in the two inflammation models, it does highlight the dynamic role of MenSCs in orchestrating the inflammatory response. For the TGC-elicited peritonitis, MenSCs face a *germ free* inflammation model mainly driven by damage-associated molecular patterns (DAMPs)^48^ and with self-resolution capacity. In contrast, the monobacterial sepsis model is perpetuated by pathogen-associated molecular patterns (PAMPs)^49^, priming different responses from MenSCs. Ultimately, all responses suggest a role for MenSCs in preventing tissue damage and promoting homeostasis.

MenSCs administration significantly enhanced peritoneal bacterial load and bacterial dissemination to spleen and mesenteric nodes suggesting a poor control of bacterial replication and a potential inability to effectively clear this pathogen^37^. The additional in vitro experiments performed with human THP-1_PMA_ cells provided further understanding of the effect of MenSCs on macrophage functional properties. Interestingly, when co-cultured with MenSCs, THP-1_PMA_ cells experienced a time-dependent increase in bacterial internalization. This macrophage enhanced phagocytic activity has also been detected using MSCs from other tissue sources^44,45,50^ supporting the ability of MenSCs to reprogram macrophages. The increase in CD14 surface expression further supports this phagocytic activity, as macrophages can internalise Gram-negative bacteria by a CD14-dependent mechanism^51^. In addition to the upregulation of CD14, a phenotypic switch of the THP-1_PMA_ cells after MenSCs co-culture was evidenced by the reduced expression of CD80/CD86, which are heavily involved in antigen T-cell presentation. This attenuation of antigen presentation capacity could indicate a move to a more regulatory macrophage phenotype^52^.

Phagocytic cells display a variety of killing mechanisms^53^. During *Salmonella* infection, the generation of reactive oxygen species (ROS) has been found to be critical to effectively control intracellular multiplication and survival of the different *Salmonella* strains^54–57^. In vitro, we detected a significant decrease in ROS production when THP-1_PMA_ cells were directly co-cultured with MenSCs, suggesting that the defective killing observed might be due to a diminished ROS generation in response to bacteria stimuli.

The use of MenSCs as a therapeutic strategy for sepsis has been previously outlined^26^. In 2015, Alcayaga-Miranda et al. evaluated the therapeutic effect of MenSCs in a cecal ligation and puncture (CLP) sepsis mouse model showing that MenSCs IP administered improved overall survival and rescued mice from an exacerbated inflammatory response^26^. Similarly, MenSCs injection on a LPS-induced acute lung injury mouse model attenuated lung histopathological damage and inflammation and induced IL-10 upregulation in the bronchoalveolar fluid (BALF)^24^. Moreover, a reduction in the BALF cell count was found, similar to what we observed in the mice peritoneal fluid of our sepsis model. However, there are certain key differences between these sepsis models and our own. Firstly, they both make use of C57BL6/j mice, a prototypical Th_1_-dominant mouse strain, whereas we employ BALB/c mice, a Th_2_- dominant one^58^. This specific background can condition the animal inflammatory milieu and therefore the context encountered by MenSCs when they are injected. Secondly, the mechanism of infection and multiplication differs between bacterial strains. The CLP model is based on commensal bacteria whose multiplication method is extracellular^59^ while, in our case, *Salmonella* strains cause intracellular infection^56^. In the same manuscript^26^, Alcayaga-Miranda et al. demonstrated in vitro that MenSCs showed anti-microbial activity mediated, at least in part, by the microbicidal peptide hepcidin. We agree with this direct anti-microbial effect of MenSCs, as we have shown that challenging MenSCs with *Salmonella* strains triggers their generation of ROS, as well as their phagocytic and killing capacity (data not shown). However, the reduction in the peritoneal phagocytes recruitment observed in both TGC and *S.* Typhimurium model, along with the in vitro human reprogramming of THP-1_PMA_ cells when co-cultured with MenSCs, reinforces the hypothesis of MenSCs acting as key modulators of inflammation through their direct action on innate immune cells rather than through their specific anti-microbial properties.

Although our work highlights the key role of MenSCs in modulating macrophage response during acute inflammation, there are some limitations that still need to be overcome. For instance, determining the mediators responsible for MenSCs effects as well as the molecular pathways involved in the immunomodulatory effects are pending tasks which will provide extremely valuable information for MenSCs translation to clinic. In addition, extending this study to other innate immune cell populations, such as neutrophils, will contribute to a better understanding of MenSCs inflammatory sensing properties. The relevance of this work is based on giving new evidence of MenSCs as sensors and switchers of inflammation^13^, stressing that this property can boost their therapeutic beneficial effects but must also be taken into account in future clinical applications. We believe that the data presented here will set the scene for further and thorough studies about MenSCs administration for the treatment of inflammatory diseases.

## Methods

### Ethical approval and consent to participate

All protocols and methods described in this research article were performed in accordance with the relevant guidelines and regulations of the University of Granada (UGR) and approved by the Research and Ethics Committee of the same institution. Committee’s reference number for human samples collection: 186/CEIH/2016.

Committee’s reference number for animal studies: 12/12/2016-179/143-CEEA-OH-2015 and 12/12/2016-179 JJAA.

### MenSCs isolation and culture

Menstrual blood samples were obtained from 15 volunteer women at ages ranged between 20 and 35 years with a regular menstrual cycle. The recruited women had no gynaecological or autoimmune disorders, claimed to be HIV and HCV negative and were not using hormonal oral contraceptives (exclusion criteria). Prior informed consent was obtained from the volunteers.

MenSCs were isolated from menstrual blood collected with menstrual cups or tampons the second day of the menstrual cycle. Mononuclear cells were separated by density gradient centrifugation with *Ficoll-Paque* (Merck KGaA, Darmstadt, Germany) and transferred into Opti-MEM media supplemented with 1% penicillin–streptomycin, amphotericin B and 3% heat-inactivated fetal bovine serum (FBS) (all from Thermo Fisher Scientific, Waltham MA, USA). MenSCs were cultured at 37 °C in a 5% CO_2_ humidified atmosphere with media changes every 3–4 days. All the experiments were performed using MenSCs at early passages (P2-P6).

MenSCs optical images were taken by a Leica microscope (DM IL LED, Leica Camera AG, Wetzlar, Germany) equipped with a charge-coupled device (CCD) camera.

### THP-1 cell culture and differentiation

THP-1 cells were grown in RPMI 1640 (GE Healthcare, Chicago, IL, USA) media supplemented with 1 mM Sodium Pyruvate, 2 mM l-Glutamine, 25 mM HEPES buffer and 10% heat-inactivated FBS (all from Thermo Fisher Scientific). The cells were cultured at 37 °C in a 5% CO_2_ humidified atmosphere with media changes every 3–4 days. In order to differentiate them to naïve macrophages, THP-1 cells were incubated with 10 ng/ml phorbol 12-myristate 13-acetate (PMA, Merck) for 24 h and cultured in Opti-MEM media supplemented with 1% penicillin–streptomycin, amphotericin B and 3% heat-inactivated FBS for additional 24 h prior to their use.

For phenotype assessment studies, 4 × 10^5^ differentiated THP-1 cells/well were cultured in a 6-well plate with Opti-MEM supplemented media and a 0.4-μm-pore size *Corning* Transwell inserts (Merck) on top of the plate, where 1 × 10^5^ MenSCs were seeded (ratio 4:1). The Transwell system was incubated for further 72 h. PMA-differentiated THP-1 cells without co-culture were used as a control.

### Bacterial strains

Wild type *Salmonella enterica* subsp. *enterica* serovar Typhimurium (NCTC 12023) and attenuated resistant to ampicillin *Salmonella enterica* subsp. *enterica SseB*^*−*^ (MvP643, p3232) strains were routinely grown overnight under sterile conditions in Luria–Bertani (LB) broth and LB with 100 µg/ml of ampicillin, respectively. Both bacterial strains were kindly donated by Dr. Michael Hensel (University of Osnabrück, Germany). When required, estimation of bacterial concentration was measured using a spectrophotometer (Spectronic Genesys 8, Spectronic Instruments).

### Animals

Eight-to ten-week-old female BALB/c mice were group-housed at the Animal Facility of Centro de Investigación Biomédica (UGR) in stable humidity and temperature conditions on a 12:12-h light/dark cycle, with free access to food and water.

### TGC-elicited peritonitis mouse model

In order to establish acute sterile peritonitis, 4% fluid TGC medium (Becton, Dickinson and Company (BD), Franklin Lakes, NJ, USA) was administered via intraperitoneal (IP) injection. Four or 24 h later, 2 × 10^6^ MenSCs were also IP administered. A single injection of TGC or phosphate-buffered saline (PBS) administration were used as control groups and single administration of MenSCs was also evaluated.

Mice were euthanized by CO_2_ inhalation 4 days after TGC injection in order to collect peritoneal exudates. Briefly, 8 ml of ice-cold PBS were IP administered and the exudates were harvested after 1 min of peritoneal massage. This process was repeated up to three times, collecting a total volume of 24 ml. After centrifugation at 1500 rpm for 5 min, the cell pellets were resuspended in PBS, counted using a hemocytometer (ZMB007, Zuzi, Spain) and supplemented with 0.5% bovine serum albumin (BSA)-2 mM EDTA for flow cytometry assessment.

### Monobacterial sepsis model

A model of systemic typhoid-like disease was generated by infecting mice with *Salmonella enterica* subsp. *enterica* serovar Typhimurium (*Salmonella*) strains. Since the WT *Salmonella* strain (ST) cannot by eliminated by mice, its injection is accompanied by the *Salmonella* attenuated strain *SseB*^*−*^ (*SseB*^*−*^*),* which lacks a pathogenicity island 2 loci thus resulting in attenuated virulence and defective intracellular surviving^35^, allowing to monitor the course of the infection resolution. Hence, mice were IP challenged with 2.5 × 10^4^ ST + 2.5 × 10^4^ *SseB*^*−*^ bacteria of *Salmonella* strains and four hours later, coincident with an acute phase of the disease, 2·10^6^ MenSCs were also IP administered. A single injection of both *Salmonella* strains was used as an infected control group.

Mice were euthanized by CO_2_ inhalation 24 h after infection. Peritoneal exudates were collected to characterise the phenotype of cells recruited as described above. Mice’s spleen and mesenteric nodes were homogenized in PBS and plated in LB and LB + ampicillin (100 µg/ml) agar plates under sterile conditions to determine the bacterial load by counting the number of colonies. Peritoneal fluid bacterial burden was also analysed.

Body weight was monitored prior to *Salmonella* injection and before sacrifice. Spleen weight was also measured and relativised to the mice body weight before sacrifice.

### Flow cytometry analysis

Phenotype assessment of MenSCs and PMA-differentiated THP-1 cells was carried out by flow cytometry using direct or indirect staining with a panel of antibodies (Table S2). Briefly, cells were washed with PBS supplemented with 0.5% BSA and 2 mM EDTA prior to incubation with the antibodies of interest or the proper isotype controls and secondary antibodies where appropriate. PMA-differentiated THP-1 cells had an extra step with *Fc-block* (anti-CD16/CD32, BD) for 15 min prior to surface markers staining. After a 30-min incubation at 4 °C, samples were characterised on a FACSCalibur (BD) flow cytometer and analysed using *FlowJo software* (v.10, FlowJo LLC).

Regarding the animal studies, the peritoneal lavage cells were also incubated with *Fc-block* for 15 min prior to surface markers direct staining (Table S2). Appropriate isotype controls were also used. After 30 min incubation at 4 °C, samples were acquired on a FACSCanto II flow cytometer and analysed using *FACSDiva software* (BD). Gates set on forward and side angle light scatter were used to exclude dead cells and debris and the number of positive cells was calculated as [(%) positive cells x total cell count]/100.

### Confocal microscopy and immunohistochemistry

Peritoneal aggregates were collected from the peritoneal cavity and snap-frozen in an OCT-filled mold (*Tissue-Tek O.C.T Compound*, Sakura SI Co. Ltd., Tokyo, Japan) immersed in isopentane on a liquid nitrogen-cooled metal surface. Samples were stored at − 80 °C until the cryosections were performed.

For confocal microscopy analysis, 7-μm thick cryosections were fixed in 3.7% paraformaldehyde (PFA). After rehydration with PBS, sections were blocked for 45 min at room temperature in PBS-Tween (0.1%)-BSA (5%) containing goat serum (5%) and Triton X-100 (0.2%). Then, slides were stained using anti-mouse F4/80, Ly-6G and anti-human nuclei (Table S2) overnight at 4 °C. After three washes with PBS-Tween (0.1%), sections were incubated where appropriate with secondary antibodies (Table S2) for 2 h at room temperature. The slides were counterstained with Hoechst 33258 (Merck) and mounted in *Fluoroshield* mounting media (Merck).

Confocal image acquisition was performed using a Nikon A1 confocal microscope (Nikon Instruments Inc., Tokyo, Japan) and the images were processed with the *NIS-Elements AR 3.2* imaging software.

For the histopathological analysis, the peritoneal aggregates were embedded in paraffin, sectioned with a microtome, and stained with hematoxilin and eosin (H&E) solution. Histology analyses were assigned in a blinded fashion by a pathologist. The identification of human cells was performed using a human cytokeratin CAM 5.2 antibody labelled with peroxidase.

### Quantitative PCR

Total RNA was extracted from peritoneal exudates using the TRIzol reagent (Thermo Fisher Scientific). cDNA was synthesized using *Access RT-PCR System kit* (Promega Corporation, Madison, WI, USA) with oligo-dT primers and according to the manufacturer’s protocol.

For quantitative PCR, appropriate primers and *FastStart Universal SYBR Green Master* (Merck) were used according to the manufacturer’s instructions on a *Quantstudio 3 Real-time PCR* system (Applied Biosystems, Foster City, CA, USA).

Data were normalized to the expression of the housekeeping gene β-actin (*Actb*) and shown as fold change relative to mRNA expression levels in infected mice without MenSCs injection applying the 2^− ΔCT^ formula.

Primer sequences are listed in Table S3.

### Phagocytosis assay

To determine the phagocytic activity of PMA-differentiated THP-1 cells, 4 × 10^5^ differentiated THP-1 cells/well were cultured in a 6-well plate with antibiotic-free Opti-MEM (3% FBS) and 0.4-μm-pore size *Corning* Transwell inserts (Merck) on top of the plate, where 1 × 10^5^ MenSCs were seeded (ratio 4:1). The Transwell system was incubated for further 48 h. PMA-differentiated THP-1 cells without co-culture were used as a control.

After co-culture, the Transwell inserts containing MenSCs were discarded and *Salmonella* Typhimurium *SseB*^*−*^ was added to the THP-1 cells monolayer at a multiplicity of infection (MOI) of 25. Plates were centrifuged for 1 min at 3500 rpm to synchronize bacterial uptake and incubated at 37 °C for 30 or 60 min. After the indicated times, cells were washed three times with PBS to remove extracellular bacteria and lysed with 1 ml of 0.5% Triton X-100 in miliQ water for 10 min at room temperature. Lysates were serially diluted and incubated in LB + ampicillin (100 µg/ml) agar plates overnight at 37ºC and the number of colonies was counted.

### Gentamicin protection assay

Killing activity of PMA-differentiated THP-1 cells was measured using a gentamicin protection assay^60^. Briefly, 4 × 10^5^ differentiated THP-1 cells/well were co-cultured or not with 1 × 10^5^ MenSCs/well using a Transwell system as described above. After 48 h of co-culture, THP-1 cells were challenged with *Salmonella* Typhimurium *SseB*^*−*^ at a MOI of 25 for 1 h at 37 °C. Once infection took place, cells were washed twice with PBS and the medium was supplemented with 100 µg/ml gentamicin (Thermo Fisher Scientific) for 1 h to kill extracellular bacteria. Thereafter, fresh medium containing 10 µg/ml gentamicin was added and kept for 6, 24 and 48 h at 37 °C. At the indicated time points, cells were washed twice with PBS and lysed with 1 ml of 0.5% Triton X-100 in miliQ water for 10 min at room temperature. Serial dilutions of the lysates were incubated in LB + ampicillin (100 µg/ml) agar plates overnight at 37 °C and colony-forming units (CFU) were counted. Cells fixed in PFA before the bacterial challenge were used as a killing negative control.

### Intracellular ROS detection

Intracellular H_2_O_2_ generation was measured using 2′,7-Dichlorofluorescin Diacetate (DCFDA, Merck) as previously described^54,61^. Briefly, PMA-differentiated THP-1 cells were cultured in a dark—clear bottom—96-well microplate (5 × 10^4^ cells/well) with antibiotic-free Opti-MEM (3% FBS) and 1.25 × 10^4^ MenSCs were directly seeded (ratio 4:1) continuing to be cultivated for an additional 48 h. PMA-differentiated THP-1 cells and MenSCs without co-culture were used as controls.

After incubation, cells were stained in the diluted DCFDA solution (3 μM) at 37 °C for 1 h in the dark and then washed twice with PBS. Next, cells were stimulated with *Salmonella* Typhimurium *SseB*^*−*^ at a MOI of 100 and the fluorescence intensity was measured at different times in a plate reader (Synergy Neo2, Biotek Instruments Inc., Winooski, VT, USA) with an excitation wavelength and emission wavelength set of 485 nm and 535 nm respectively.

Results are expressed as fluorescent relative units (RU) after blank subtraction (DCFDA-unstimulated cells).

### Statistical analysis

Results are expressed as the mean ± SEM. For the TGC-elicited peritonitis mouse model, statistical differences were determined using ordinary one or two-way ANOVA with Tukey’s multiple comparison post hoc test. N is ranged between 4 and 9 mice per experimental group.

Regarding the monobacterial sepsis model, the unpaired two-tailed Student’s *t*-test was used for body weight loss, spleen weight and total cell count whereas two-way ANOVA with Sidak’s multiple comparison post hoc test was used for phenotypic analysis, CFU quantification and mRNA expression levels. N is ranged between 12 and 13 mice per experimental group.

For the in vitro assays statistical differences were determined using two-way ANOVA with Tukey’s (Phagocytosis, ROS production) or Sidak’s (Gentamicin protection assay, THP-1 phenotype assessment) post hoc tests. In this case, the number of replicates ranged from 3–10, using a minimum of 3 different MenSCs donors.

All the statistical analyses were performed using *GraphPad Prism 6* (GraphPad Software). P values less than 0.05 were interpreted as statistically significant and represented as *p < 0.05, **p < 0.01, ***p < 0.001, ****p < 0.0001.

## Supplementary Information

Supplementary Information.

## Data Availability

All data generated or analysed during this study are included in this published article and its supplementary information files.

## References

[1] Meng X (2007). Endometrial regenerative cells: a novel stem cell population. J. Transl. Med..

[2] Alcayaga-Miranda F (2015). Characterization of menstrual stem cells: angiogenic effect, migration and hematopoietic stem cell support in comparison with bone marrow mesenchymal stem cells. Stem Cell Res. Ther..

[3] Polymeri A, Giannobile W, Kaigler D (2016). Bone marrow stromal stem cells in tissue engineering and regenerative medicine. Horm. Metab. Res..

[4] Patrikoski M, Mannerström B, Miettinen S (2019). Perspectives for clinical translation of adipose stromal/stem cells. Stem Cells Int..

[5] Batsali K, Kastrinaki M-C, Papadaki H, Pontikoglou C (2013). Mesenchymal stem cells derived from Wharton’s jelly of the umbilical cord: biological properties and emerging clinical applications. Curr. Stem Cell Res. Ther..

[6] Roura S, Pujal J-M, Gálvez-Montón C, Bayes-Genis A (2015). The role and potential of umbilical cord blood in an era of new therapies: a review. Stem Cell Res. Ther..

[7] Muñoz-Fernández R (2019). Human predecidual stromal cells are mesenchymal stromal/stem cells and have a therapeutic effect in an immune-based mouse model of recurrent spontaneous abortion. Stem Cell Res. Ther..

[8] Mathew SA, Naik C, Cahill PA, Bhonde RR (2020). Placental mesenchymal stromal cells as an alternative tool for therapeutic angiogenesis. Cell. Mol. Life Sci..

[9] Bozorgmehr, M. *et al.* Endometrial and menstrual blood mesenchymal stem/stromal cells: biological properties and clinical application. *Front. Cell Dev. Biol.* **8**, (2020). 10.3389/fcell.2020.00497 PMC7364758 32742977

[10] Rodrigues MCO (2016). Menstrual blood-derived stem cells: in vitro and in vivo characterization of functional effects. Adv. Exp. Med. Biol..

[11] Uccelli A, Moretta L, Pistoia V (2008). Mesenchymal stem cells in health and disease. Nat. Rev. Immunol..

[12] Zhou Y, Yamamoto Y, Xiao Z, Ochiya T (2019). The immunomodulatory functions of mesenchymal stromal/stem cells mediated via paracrine activity. J. Clin. Med..

[13] Bernardo ME (2013). Mesenchymal stromal cells: Sensors and switchers of inflammation. Cell Stem Cell.

[14] Le Blanc K, Davies LC (2015). Mesenchymal stromal cells and the innate immune response. Immunol. Lett..

[15] Elman JS, Li M, Wang F, Gimble JM, Parekkadan B (2014). A comparison of adipose and bone marrow-derived mesenchymal stromal cell secreted factors in the treatment of systemic inflammation. J. Inflamm..

[16] Shi Y (2018). Immunoregulatory mechanisms of mesenchymal stem and stromal cells in inflammatory diseases. Nat. Rev. Nephrol..

[17] de Castro LL, Lopes-Pacheco M, Weiss DJ, Cruz FF, Rocco PRM (2019). Current understanding of the immunosuppressive properties of mesenchymal stromal cells. J. Mol. Med..

[18] Mattar P, Bieback K (2015). Comparing the immunomodulatory properties of bone marrow, adipose tissue, and birth-associated tissue mesenchymal stromal cells. Front. Immunol..

[19] Ren H, Zhang Q, Wang J, Pan R (2018). Comparative effects of umbilical cord- and menstrual blood-derived MSCs in repairing acute lung injury. Stem Cells Int..

[20] Fathi-Kazerooni M (2017). Comparative restoration of acute liver failure by menstrual blood stem cells compared with bone marrow stem cells in mice model. Cytotherapy.

[21] Cuenca J (2018). The reparative abilities of menstrual stem cells modulate the wound matrix signals and improve cutaneous regeneration. Front. Physiol..

[22] Lv Y (2014). Endometrial regenerative cells as a novel cell therapy attenuate experimental colitis in mice. J. Transl. Med..

[23] Xu X (2018). Treatment of experimental colitis by endometrial regenerative cells through regulation of B lymphocytes in mice. Stem Cell Res. Ther..

[24] Xiang B (2017). Transplantation of menstrual blood-derived mesenchymal stem cells promotes the repair of LPS-induced acute lung injury. Int. J. Mol. Sci..

[25] Jin W (2020). Stromal cell-derived factor-1 enhances the therapeutic effects of human endometrial regenerative cells in a mouse sepsis model. Stem Cells Int..

[26] Alcayaga-Miranda F (2015). Combination therapy of menstrual derived mesenchymal stem cells and antibiotics ameliorates survival in sepsis. Stem Cell Res. Ther..

[27] Fujiwara N, Kobayashi K (2005). Macrophages in Inflammation. Curr. Drug Target Inflam. Allergy.

[28] Ruiz Magaña, M. J. *et al.* Endometrial and decidual stromal precursors show a different decidualization capacity. *Reproduction* **160**, 83–91 (2020). 10.1530/REP-19-0465 32422602

[29] Dominici, M. *et al.* Minimal criteria for defining multipotent mesenchymal stromal cells. The International Society for Cellular Therapy position statement. *Cytotherapy* **8**, 315–317 (2006). 10.1080/14653240600855905 16923606

[30] Wang G (2012). Cutting Edge: Slamf8 is a negative regulator of Nox2 activity in macrophages. J. Immunol..

[31] Leijh PC, van Zwet TL, ter Kuile MN, van Furth R (1984). Effect of thioglycolate on phagocytic and microbicidal activities of peritoneal macrophages. Infect. Immun..

[32] Baron EJ, Proctor RA (1982). Elicitation of peritoneal polymorphonuclear neutrophils from mice. J. Immunol. Methods.

[33] Lam D, Harris D, Qin Z (2013). Inflammatory mediator profiling reveals immune properties of chemotactic gradients and macrophage mediator production inhibition during thioglycollate elicited peritoneal inflammation. Mediat. Inflamm..

[34] Poli-de-Figueiredo LF, Garrido AG, Nakagawa N, Sannomiya P (2008). Experimental models of sepsis and their clinical relevance. Shock.

[35] Hölzer SU, Hensel M (2010). Functional dissection of translocon proteins of the Salmonella Pathogenicity Island 2-encoded type III secretion system. BMC Microbiol..

[36] Keane C, Jerkic M, Laffey JG (2017). Stem cell–based therapies for sepsis. Anesthesiology.

[37] Brawn LC, Hayward RD, Koronakis V (2007). Salmonella SPI1 effector SipA persists after entry and cooperates with a SPI2 effector to regulate phagosome maturation and intracellular replication. Cell Host Microbe.

[38] Sala E (2015). Mesenchymal stem cells reduce colitis in mice via release of TSG6, independently of their localization to the intestine. Gastroenterology.

[39] Wu X (2014). Transplantation of human menstrual blood progenitor cells improves hyperglycemia by promoting endogenous progenitor differentiation in type 1 diabetic mice. Stem Cells Dev..

[40] Khanjani S (2015). Efficient generation of functional hepatocyte-like cells from menstrual blood-derived stem cells. J. Tissue Eng. Regen. Med..

[41] Chen L (2017). Human menstrual blood-derived stem cells ameliorate liver fibrosis in mice by targeting hepatic stellate cells via paracrine mediators. Stem Cells Transl. Med..

[42] Bystrom J (2008). Resolution-phase macrophages possess a unique inflammatory phenotype that is controlled by cAMP. Blood.

[43] Anderson P (2013). Adipose-derived mesenchymal stromal cells induce immunomodulatory macrophages which protect from experimental colitis and sepsis. Gut.

[44] Maggini J (2010). Mouse bone marrow-derived mesenchymal stromal cells turn activated macrophages into a regulatory-like profile. PLoS ONE.

[45] Kim J, Hematti P (2009). Mesenchymal stem cell–educated macrophages: A novel type of alternatively activated macrophages. Exp. Hematol..

[46] Fleming SD, Campbell PA (1996). Macrophages have cell surface IL-10 that regulates macrophage bactericidal activity. J. Immunol..

[47] Németh K (2009). Bone marrow stromal cells attenuate sepsis via prostaglandin E2-dependent reprogramming of host macrophages to increase their interleukin-10 production. Nat. Med..

[48] Chen GY, Nuñez G (2010). Sterile inflammation: sensing and reacting to damage. Nat. Rev. Immunol..

[49] Rajaee A, Barnett R, Cheadle WG (2018). Pathogen- and danger-associated molecular patterns and the cytokine response in sepsis. Surg. Infect. (Larchmt).

[50] Zhang QZ (2010). Human gingiva-derived mesenchymal stem cells elicit polarization of M2 macrophages and enhance cutaneous wound healing. Stem Cells.

[51] Grunwald U (1996). Monocytes can phagocytose Gram-negative bacteria by a CD14-dependent mechanism. J. Immunol..

[52] Vasandan AB (2016). Human Mesenchymal stem cells program macrophage plasticity by altering their metabolic status via a PGE 2 -dependent mechanism. Sci. Rep..

[53] Srikanth CV, Cherayil BJ (2007). Intestinal innate immunity and the pathogenesis of Salmonella enteritis. Immunol. Res..

[54] Li Y (2017). P40phox-deficient mice exhibit impaired bacterial clearance and enhanced pro-inflammatory responses during Salmonella enterica serovar Typhimurium infection. Front. Immunol..

[55] Taub N (2012). The late endosomal adaptor p14 is a macrophage host-defense factor against Salmonella infection. J. Cell Sci..

[56] Mastroeni, P. *et al.* Antimicrobial actions of the NADPH phagocyte oxidase and inducible nitric oxide synthase in experimental salmonellosis. II. Effects on microbial proliferation and host survival in vivo. *J. Exp. Med.* **192**, 237–248 (2000). 10.1084/jem.192.2.237 PMC2193252 10899910

[57] Vazquez-Torres A (2000). Salmonella pathogenicity island 2-dependent evasion of the phagocyte NADPH oxidase. Science.

[58] Watanabe H, Numata K, Ito T, Takagi K, Matsukawa A (2004). Innate immune response in Th1- and Th2-dominant mouse strains. Shock.

[59] Ruiz S (2016). Sepsis modeling in mice: ligation length is a major severity factor in cecal ligation and puncture. Intensive Care Med. Exp..

[60] Laroux FS, Romero X, Wetzler L, Engel P, Terhorst C (2005). Cutting edge: MyD88 controls phagocyte NADPH oxidase function and killing of gram-negative bacteria. J. Immunol..

[61] El Bekay R (2002). Activation of phagocytic cell NADPH oxidase by norfloxacin: a potential mechanism to explain its bactericidal action. J. Leukoc. Biol..

