# Supplementary material for: Menstrual blood-derived stromal cells modulate functional properties of mouse and human macrophages
Source: Sci Rep. 2020 Dec 7;10:21389. doi: 10.1038/s41598-020-78423-x (PMC7721726; doi:10.1038/s41598-020-78423-x)
Supplement: Supplementary file 1 — Supplementary Information. [file 41598_2020_78423_MOESM1_ESM.pdf]

SUPPLEMENTARY INFORMATION

Menstrual blood-derived stromal cells modulate functional properties of mouse and human macrophages

Rocío Martínez-Aguilar[1], Salvador Romero-Pinedo[1], M. José Ruiz-Magaña[1], Enrique G. Olivares[2,3], Carmen Ruiz-Ruiz[1,2†*] and Ana C. Abadía-Molina[1,2†*]

*Corresponding author: mcarmenr@ugr.es, acbadia@ugr.es.

† Ana C. Abadía-Molina and Carmen Ruiz-Ruiz jointly supervised this work.

[1] Unidad de Inmunología, IBIMER, CIBM, Universidad de Granada, Granada, Spain.

[2] Departamento de Bioquímica y Biología Molecular III e Inmunología, Facultad de Medicina, Universidad de Granada, Granada, Spain.

[3] Unidad de Gestión Clínica Laboratorios, Hospital Universitario Clínico San Cecilio, Granada, Spain.

Supplementary figure 1. Immunophenotypic characterisation of MenSCs. Fibroblast-like morphology of freshly isolated (**a**) and crystal violet-stained (**b**) MenSCs. Images were taken with objective lens at 4x magnification. (**c**) Phenotype of MenSCs from three different donors determined by flow cytometry. Mean ± SEM (N=3) values are depicted, where grey histograms refer to adequate isotype control.

Supplementary table 1. Statistical significance of the ST and *SseB*$^{-}$ bacterial load in spleen, peritoneal fluid and mesenteric nodes from infected mice treated with and without MenSCs.

Supplementary table 2. List of antibodies used for phenotypic characterisation.

Supplementary table 3. List of primers.

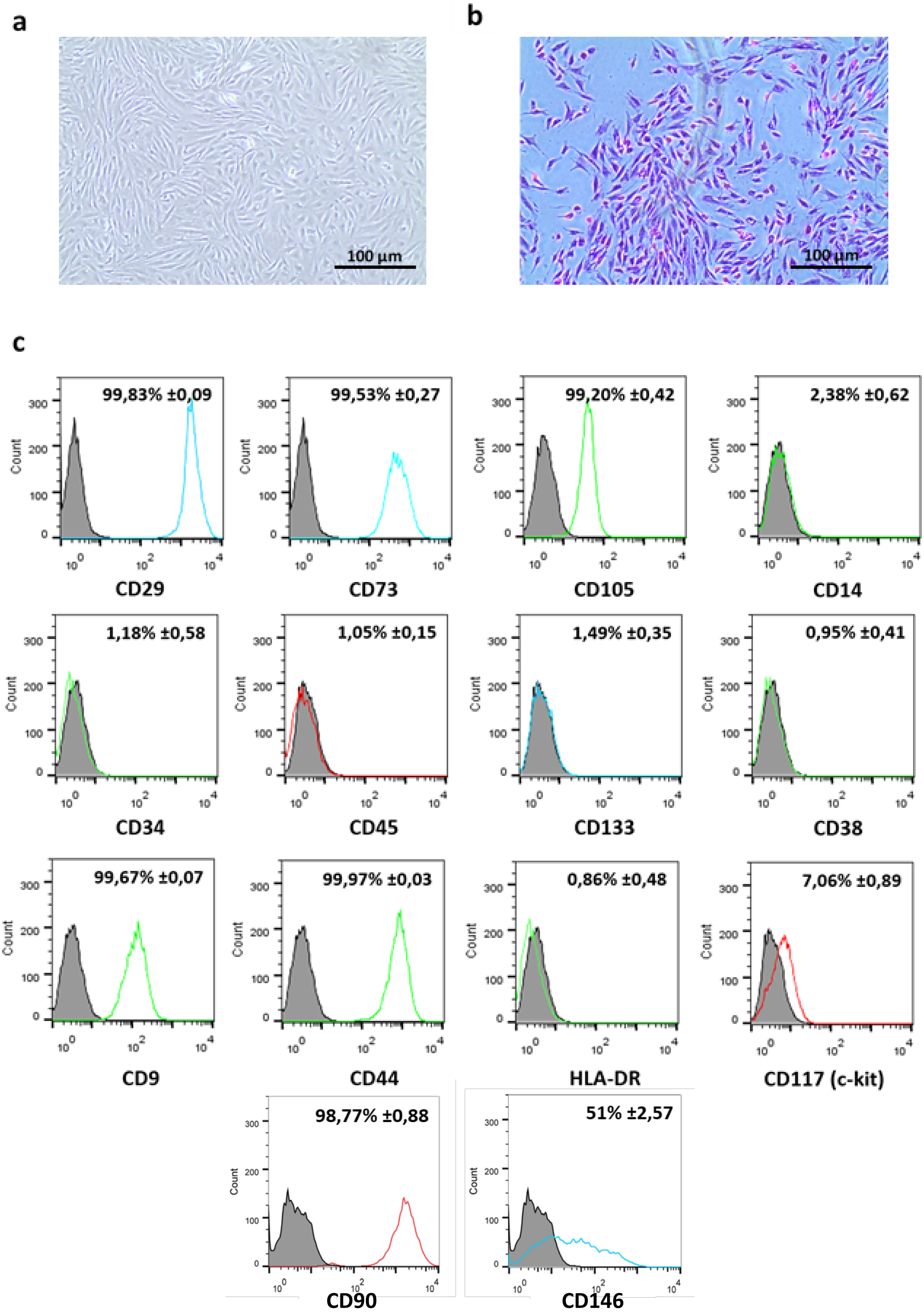

SUPPLEMENTARY FIGURE 1 (S1)

			Mean ± SEM	p-value
**Spleen**	**ST**	**- MenSCs**	$1.6 \cdot 10^5 \pm 1.2 \cdot 10^4$	p<0.0001
		**+ MenSCs**	$8.7 \cdot 10^5 \pm 7.9 \cdot 10^4$	
	SseB⁻	**- MenSCs**	$8.7 \cdot 10^4 \pm 5.9 \cdot 10^3$	p<0.0001
		**+ MenSCs**	$5.5 \cdot 10^5 \pm 5.2 \cdot 10^4$	
**Peritoneal fluid**	**ST**	**- MenSCs**	$1.2 \cdot 10^5 \pm 1.8 \cdot 10^4$	p<0.0001
		**+ MenSCs**	$4 \cdot 10^5 \pm 3.3 \cdot 10^4$	
	SseB⁻	**- MenSCs**	$7.6 \cdot 10^4 \pm 5 \cdot 10^3$	p<0.0001
		**+ MenSCs**	$3.9 \cdot 10^5 \pm 1.6 \cdot 10^4$	
**Mesenteric nodes**	**ST**	**- MenSCs**	$7.6 \cdot 10^3 \pm 1.2 \cdot 10^3$	p<0.0001
		**+ MenSCs**	$8.1 \cdot 10^4 \pm 1.5 \cdot 10^4$	
	SseB⁻	**- MenSCs**	$4.1 \cdot 10^3 \pm 7.4 \cdot 10^2$	p<0.01
		**+ MenSCs**	$4.9 \cdot 10^4 \pm 7 \cdot 10^3$	

SUPPLEMENTARY TABLE 1 (TABLE S1)

Antibody	Fluorochrome	Clone	Manufacturer
**Flow Cytometry**			
Anti-human CD9	FITC	HI9a	Biolegend
Anti-human CD14	FITC	HCD14	Biolegend
Anti-human CD29	APC	TS2/16	Biolegend
Anti-human CD34	FITC	581	Biolegend
Anti-human CD38	FITC	HIT2	Biolegend
Anti-human CD44	FITC	BJ18	Biolegend
Anti-human CD45	PE	HI30	Biolegend
Anti-human CD73	APC	AD2	Miltenyi Biotec
Anti-human CD90	PE	5E10	Biolegend
Anti-human CD105	-	P4A4	Gift from Dr F.J. Blanco (UGR)
Anti-human CD117 (c-kit)	PE	104D2	Biolegend
Anti-human CD133	APC	REA816	Miltenyi Biotec
Anti-human CD146	APC	P1H12	Biolegend
Anti-human HLA-DR	FITC	REA805	Miltenyi Biotec
Anti-human CD11b	FITC	ICRF44	Biolegend
Anti-human CD14	APC	M5E2	Biolegend
Anti-human CD16	PE	3G8	Biolegend
Anti-human CD80	PE	2D10	Biolegend
Anti-human CD86	APC	IT2.2	Biolegend
Mouse IgG1, κ Isotype Control	APC	P1H12	Biolegend
Mouse IgG1, κ Isotype Control	FITC	MOPC-21	Biolegend
Mouse IgG1, κ Isotype Control	PE	MOPC-21	Biolegend
F(ab')$_2$ goat Anti-mouse IgG (H+L)	AF 488	Polyclonal	Invitrogen
Anti-mouse B220	PerCP-Cy5.5	RA3-6B2	eBioscience
Anti-mouse CD3e	FITC	145-2C11	eBioscience
Anti-mouse/human CD11b	APC/Cy7	M1/70	Biolegend
Anti-mouse CD11c	FITC	N418	eBioscience
Anti-mouse F4/80	APC	BM8	Biolegend
Anti-mouse Ly-6G (Gr-1)	PE	RB6-8C5	eBioscience
Anti-mouse Ly-6C	FITC	AL-21	BD Biosciences
Rat IgM, κ Isotype Control	FITC	R4-22	BD Pharmingen
Rat IgG2a, κ Isotype Control	PE	KLH/G2a-1-1	Southern Biotech
Rat IgG2b, κ Isotype Control	APC	RTK4530	Biolegend
Rat IgG2b, κ Isotype Control	APC/Cy7	RTK4530	Biolegend
Rat IgG2a, κ Isotype Control	PerCP-Cy5.5	eBR2A	eBioscience
**Confocal Microscopy**			
Anti-Mouse F4/80	-	Cl:A3-1	BIO-RAD
Anti-Mouse Ly-6G	-	1A8	BD Pharmingen
Anti-Human nuclei	AF488	235-1	Merck
Goat Anti-Rat IgG (H+L)	AF488	Polyclonal	Thermo Fisher Scientific
Goat Anti-Rat IgG (H+L)	AF594	Polyclonal	Thermo Fisher Scientific

SUPPLEMENTARY TABLE 2 (TABLE S2)

Gene	Primer sequence	Product size (bp)	Tm (ºC)
**iNOS**	**F:** 5′-ATT CTT GGA GCG AGT TGT GG-3′<br>**R:** 5′-CAG GAA GTA GGT GAG GGC TTG-3′	139	59
**IL-10**	**F:** 5′-GAA GAC CCT CAG GAT GCG G-3′<br>**R:** 5′-CCT GCT CCA CTG CCT TGC T-3′	77	59
**TGF-β**	**F:** 5′-TGA CGT CAC TGG AGT TGT ACG G-3′<br>**R:** 5′-GGT TCA TGT CAT GGA TGG TGC-3′	170	59
**TNF-α**	**F:** 5′- CAC AAG ATG CTG GGA CAG TGA-3′<br>**R:** 5′-TCC TTG ATG GTG GTG CAT GA-3′	58	55
**β-actin**	**F:** 5′-TGT TAC CAA CTG GGA CGA CA-3′<br>**R:** 5′- GGG GTG TTG AAG GTC TCA AA-3′	165	59

SUPPLEMENTARY TABLE 3 (TABLE S3)
